# Requirement of Leukemia Inhibitory Factor or Epidermal Growth Factor for Pre-Implantation Embryogenesis via JAK/STAT3 Signaling Pathways

**DOI:** 10.1371/journal.pone.0153086

**Published:** 2016-04-20

**Authors:** En-Hui Cheng, Jer-Yuh Liu, Tsung-Hsein Lee, Chun-Chia Huang, Chung-I Chen, Lii-Sheng Huang, Maw-Sheng Lee

**Affiliations:** 1 Division of Infertility Clinic, Lee Women’s Hospital, Taichung, Taiwan; 2 Graduate Institute of Cancer Biology, China Medical University, Taichung, Taiwan; 3 Center for Molecular Medicine, China Medical University Hospital, Taichung, Taiwan; 4 Department of Obstetrics and Gynecology, Chung Shan Medical University Hospital, Taichung, Taiwan; 5 Institute of Medicine, Chung Shan Medical University, Taichung, Taiwan; 6 Department of Nursing, School of Medicine, Chung Shan Medical University, Taichung, Taiwan; University of Kansas Medical Center, UNITED STATES

## Abstract

Leukemia inhibitory factor (LIF) plays a key role in the survivability of mouse embryos during pre-implantation. In this study, we verified the role of LIF by detecting gene expression in morula stage embryos through DNA microarray. Our results showed that LIF knockdown affected expression of 369 genes. After LIF supplementation, the epidermal growth factor (EGF) is most affected by LIF expression. To observe the correlation between LIF and EGF, the LIF knockdown embryos were supplemented with various growth factors, including LIF, EGF, GM-CSF, TGF, and IGF II. Only LIF and EGF caused the rate of blastocyst development to recover significantly from 52% of control to 83% and 93%, respectively. All of the variables, including the diameter of blastocysts, the number of blastomeres, and cells in ICM and TE, were almost restored. Moreover, EGF knockdown also impaired blastocyst development, which was reversed by LIF or EGF supplementation. The treatment with various signaling suppressors revealed that both EGF and LIF promoted embryonic development through the JAK/STAT3 signaling pathway. These data suggest that the EGF and LIF can be compensatory to each other during early embryonic development, and at least one of them is necessary for sustaining the normal development of pre-implantation embryos.

## Introduction

Leukemia inhibitory factor (LIF) is a cytokine that regulates the survival, differentiation, and proliferation of various cells, both in adults and in embryos [1–4]. LIF is involved in various reproductive processes, including sperm enhancement, regulation of ovulation, as well as blastocyst formation, hatching, and implantation in embryos [5–8].

An experiment that co-cultured mouse embryos with human oviductal cells to produce a cocktail of embryo nourishing factors, including LIF, resulted in maintained mitochondrial function, decreased apoptosis in the embryo, and a higher rate of blastocyst formation compared with those in mouse embryos without co-culture [9–11]. Several reports also support the effect of LIF supplementation on blastocyst formation [12–15]. Our previous study also showed that LIF deficiency can significantly decrease the survivability of embryos during four-cell, morula, and blastocyst development [16]. These findings strongly suggest that LIF is a critical factor for normal development of embryos in the pre-implantation stages. Although the role of LIF during implantation has been widely investigated, the mechanism through which it functions during pre-implantation embryogenesis remains unclear.

LIF exerts its biological effects by interaction through its specific LIF receptor (LIFR) [17]. Expression of LIFR has been demonstrated in all pre-embryonic stages from the 2-cell stage to the expanded blastocysts [18]. LIF binds to the LIFR and can react with glycoprotein 130 (gp130), which are both situated on a receptor complex known to activate the Janus kinase/signal transducer and activator of transcription 3 (JAK/STAT3), mitogen-activated protein kinase (MAPK), and phosphatidylinositol-3 kinase (PI3K) signaling pathways [19]. Furthermore, STAT3-deficient mouse embryos exhibit a rapid degeneration during embryogenesis [20], and the LIF-JAK-STAT3 signaling pathway sustains self-renewal in mouse embryonic stem cells [21–22], which suggests the importance of the STAT3 pathway, one of the pathways directly induced by LIF. However, the specific signaling pathway induced by LIF in pre-implantation embryo development remains unknown. Other downstream LIF pathways in pre-implantation embryo development need investigation.

In this study, DNA microarray assay was used to detect gene expression in morula stage embryos to verify the role of LIF in embryonic development, especially before implantation. Various signaling suppressors were employed to test the significant signaling pathways. The critical roles of LIF and epidermal growth factor (EGF) in modulating the JAK/STAT3 signaling pathway in embryogenesis are discussed.

## Materials and Methods

### siRNA Plasmid Construction

The sequence of the 19-nucleotide siRNA-LIF duplex from the murine LIF gene (GenBank accession no. NM_008501) corresponding to the coding regions as indicated was designed and constructed by Thermo Fisher Scientific OPEN BIOSYSTEMS, INC. CA. The sequences designed to produce hairpin RNAs identical to the oligonucleotide siRNA duplex sequences are as follows (3798.3816): TGCTGTTGACAGTGAGCGAGGTGACAAATTCCTCTTTGATTAGTGAAGCCAC; AGATGTAATCAAAGAGGAATTTGTCACCCTGCCTACTGCCTCGGA. LIF siRNA duplex sequences are as follows: sense 5’-[CCAGAUCAAGAAUCAACUG]_RNA_ [TT]_DNA_, antisense 5’-[CAGUUUGAUUCUUGAUCUGG]_RNA_ [TT]_DNA_. LIF siRNA was manufactured by MWG Biotech AG, USA. The LIF siRNA was dissolved in siMAX Universal buffer (30mM HEPES, 100 mM KCl, 1mM MgCl_2_, pH7.3) and diluted by normal saline before microinjection. EGF SiRNA was puchased from Santa Cruz Biotechnology, (CA, USA)

### Oligonucleotides

Morpholino oligonucleotides were provided by Gene Tools, LLC (Phi-lomath, OR). Preparation of the LIF antisense oligonucleotide and nonsense oligonucleotide was performed as a previously published paper [16]. The stability of these oligos was determined by injecting fluorescein isothiocyanate-labeled preparations into mouse embryo and observing the results under a fluorescence microscope.

### Animals

All mice were obtained from the National Laboratory Animal Center (Taipei, Taiwan). The mice were housed in humidity- (40–60%) and temperature- (22 ~26°C)-controlled rooms and maintained on a 12L:12D photoperiod and given food and water ad libitum. All procedures were approved by the Chung-Shan Medical University Institutional Animal Care and Use Committee and were performed in accordance with the Guiding Principles for the Care and Use of Laboratory Animals.

### Preparation of Embryos

Preparation of embryos was performed as a previously published paper [16]. Zygotes were collected from the oviducts of female mice successfully mated in the laboratory and placed into wells with fresh human tubal fluid medium. Embryos in the two-pronucleus (2PN) stage were obtained by incubating the zygotes in an atmosphere with 5% CO2 at 37°C for 4 h.

### Microinjection of siRNA or Oligonucleotides

Microinjection was performed as a previously published paper [16] with some modifications. In the experimental group, the embryos were either injected with 1pl of 1.5 fmole LIF or 1.0 fmole EGF siRNA into the cytoplasma or 1pl of 2.0 fmole oligonucleotide into the male pronucleus. The embryos injected with scrambled siRNA (1pl of 1.5 fmole), or nonsense oligonucleotide (1pl of 2.0 fmole) act as a positive control. The embryos were then incubated in an atmosphere of 5% CO2 at 37°C and monitored daily using an optical microscope.

### Immunocytochemistry

Embryos were recovered from the culture medium and freed of zona pellucida by brief exposure to acidic Tyrode solution [23]. After washing three times with phosphate-buffered saline (PBS), the embryos were placed onto microscope slides and fixed in 2% formalin for 15 min. The embryos were washed in PBS and incubated in a blocking solution (10% fetal calf serum, 0.5% Tween 20, 0.02% sodium azide in PBS) for 1 h. After incubating with an affinity-purified rabbit antipeptide antibody preparation specific to LIF (1 μg/ml) (Chemicon, VIC, Australia) at 4°C overnight, the embryos were washed with the blocking solution for 10 min.

Immunostaining was performed using the VECTASTAIN ABC kit (Vector Laboratories, Burlingame, CA). The embryos were incubated with biotinylated anti-rabbit IgG (1 μg/ml) at 25°C for 1 h. The embryos were then incubated with avidin-biotinylated horseradish peroxidase at 25°C for 1 h. After washing with TBST buffer (50 mM Tris-HCl, 0.025% Tween 20, pH 7.8) five times, the embryos were treated with 3,3′-diaminobenzidine substrate (Sigma-Aldrich Co., MO) for 20 min. The embryos were dehydrated through graded alcohol and mounted with glycerol. Results of immunostaining were observed using phase-contrast microscopy. The visible staining indicated the immunoreactive LIF protein sites. Corresponding nonspecific binding of embryos was shown by parallel incubation with the antibody preneutralized with excess antigenic peptide.

### DNA microarray

Total RNA was isolated from morula stage mouse embryos (n = 20) with LIF-siRNA injection or scrambled-siRNA injection by using TRIzol reagent (Life Technologies, Rockville, MD, USA), then loaded onto a Qiagen RNeasy column (Qiagen Inc, Valencia, CA, USA) for purification. Electrophoresis on a 1% agarose formaldehyde gel was utilized to determine the integrity of the RNA preparation. Total RNA (1.5–3 μg) was reverse transcribed with Superscript II RNase H- Reverse Transcriptase (Gibco BRL, Grand Island, NY, USA) to generate Cy5 (red) and Cy3 (green) fluorescent dye-labeled complementary DNA (cDNA) probes, respectively. The labeled probes were hybridized to Agilent Mouse Oligo Microarrays, a commercial cDNA microarray (Agilent Technologies, Taipei, Taiwan) containing 9652 immobilized cDNA fragments. Fluorescence intensities of Cy3 and Cy5 targets were measured and scanned separately using a Dig Luminescent Detection Kit (Roche, Taipei, Taiwan) and Fluor-S MAZ Multi-Imager System (Bio-Rad, Hercules, CA). Data analysis was performed using Agilent Feature extraction software Version A.6.1.1 (Agilent Technologies). The criteria for determination of differentially expressed genes were that the absolute value of the ratio of Cy5 to Cy3 was greater than two-fold (up-regulated) or less than 0.5-fold (down-regulated), and the signal value of fluorescence intensity of either Cy3 or Cy5 was greater than 1,000 intensity. The data discussed in this study have been deposited in the National Center for Biotechnology Information’s Gene Expression Omnibus and can be accessed through Gene Expression Omnibus Series accession number GSE71736 (http://www.ncbi.nlm.nih.gov/geo).

### Quantitative real-Time Reverse-Transcription-Polymerase-Chain-Reaction (RT-PCR)

Messenger RNA was extracted using a commercially available Dynabeads mRNA DIRECT Kit (Dynal Biotech ASA, Oslo, Norway). We added 120 μL of Lysis/Binding Buffer to the cumulus cells and inverted the tube repeatedly to obtain complete lysis. We transferred lysate to a vial and added 10 μL of Dynabeads Oligo (dT) 25. The beads were mixed with the sample lysate and incubated with continuous mixing for 3 to 5 minutes at room temperature to allow the poly (A) tail of the mRNA to anneal to the Oligo-dT on the beads. This vial was placed on a magnet for 2 minutes to attract the mRNA-attached beads to the bottom of the vial and then the supernatant was removed from the top of the vial. The beads/mRNA complex was washed twice with 240 μL of Washing Buffer A and once with 120 μL of Washing Buffer B at room temperature.

Messenger RNA on the beads underwent reverse transcription into single-stranded cDNA. The bead/mRNA complex was added to another vial for a final reaction volume of 30 μL: 17 μL of diethyl pyrocarbonate (DEPC)-treated water, 1 μL of RNaseOUT recombinant RNase inhibitor (40 U/μL, Invitrogen Corp., Carlsbad, CA, USA), 1 μL of dNTPs (10 mM each dATP, dGTP, dCTP and dTTP), 1 μL of Oligo (dT) 20 primer (50 μM, Invitrogen Life Tech, USA), 4 μL of 5X First-Strand Buffer (Invitrogen Corp, Carlsbad, CA, USA), 2 μL of DTT (0.1 M), and 1 μL of SuperScript III Reverse Transcriptase (200 U/μL). The reaction was carried out at 50°C for 1 hour, then at 70°C for 15 minutes, 4°C for 2 minutes, and 37°C for 20 minutes. Then the single-stranded cDNA was used for real-time PCR.

The standard used for the real-time PCR was a plasmid DNA that was previously cloned from the PCR product corresponding to β-actin cDNA (primer sequence were shown as forward-1, 5’-CATGTGCAAAGCCGGCTTC-3’, and reverse-1, 5’-ATAGGAGTCCTTCTGACCCAT-3’. The standard DNA was also amplified simultaneously with 5 or 10 times serial dilutions in duplicate. The same reaction mixture with no cDNA added was run as a negative control for each gene.

Reaction volumes of 25 μL contained 1 μL of cDNA, 12.5 μL of 2x HotSybr PCR Reaction Mix (NuStar Laboratory, USA), 5 μL of each gene-specific primer (0.2–0.5 μM, depending to different genes), and 6.5 μL sterile distilled water. The mixtures were subjected to thermal cycling using a 7300 Real-Time PCR System (Applied Biosystems, Foster City, CA, USA). The amplification program consisted of heating at 95°C for 5 min, followed by 95°C for 5 sec, a calculated temperature for 30 seconds depending on the Tm of the many primers used, and 72°C for 2 min for 50 cycles.

### Treatment of Growth Factors and Kinase Inhibitors

After the 2PN mouse embryos were injected with 1pl of 2.0 fmol LIF antisense oligonucleotide or (1.5fmole) LIF siRNA, the reproductive tract growth factors [LIF, insulin-like growth factor II (IGF-II), EGF, granulocyte macrophage colony-stimulating factor (GM-CSF), and transforming growth factor beta (TGFβ) purchased from the Company of the Sigma Aldrich] and kinase inhibitors [JAK inhibitor (JAK I-II), MEK inhibitor (PD98059), PI3K inhibitor (LY294002), and EGFR inhibitor (PD168393)] were added to the culture medium. We used the LD50 of development rate of blastocysts to be the final dosage for the inhibitors (S1 Table). These embryos were then incubated in an atmosphere of 5% CO_2_ at 37°C and monitored daily using an optical microscope.

### Differential Staining of Inner Cell Mass and Trophectoderm

Cells in the trophectoderm (TE) and inner cell mass (ICM) of the blastocysts were counted after differential staining of the nuclei as described in previous study[16]. The zona-free blastocysts were incubated for 10 min at 5°C in M16 medium (Sigma) containing 10 mM trinitrobenzenesulphonic acid, 4.0 mg/ml polyvinylpyrrolidone, and 0.015% Triton X-100. After washing in M2 medium (Sigma), the blastocysts were incubated in 0.1 mg/ml anti-dinitrophenol-BSA at 37°C for 15 min and washed three more times with the M2 medium. The blastocysts were then incubated in M2 medium containing a 1:10 dilution of guinea pig complement serum (Sigma) and 10.0 μg/ml propidium iodide (Sigma) at 37°C for 15 min and washed three times with Dulbecco PBS (Gibco). After fixing in absolute ethanol containing 22.0 μg/ml bisbenzimide (Sigma) at 5°C overnight, individual blastocysts were mounted in glycerol on microscope slides to inflate the dehydrated embryos and compressed manually before visualization by epi-fluorescence using the Nikon filter blocks UV-2A and G-2A. The trophectoderm exhibit fluorescence in red and the inner cell mass in blue; the cell numbers in the two areas of the embryos were counted.

### Statistical Analysis

Embryo development rates were expressed as percentages and compared using Chi-test. Data of the quantitative of gene expression were expressed as the mean ± standard deviation (SD) and statistical analysis was carried out using the Student t-test. All the analyses were performed using the Statistical Package for the Social Sciences (version 14.0; SPSS Inc, Chicago, IL). A confidence level of P < 0.05 was considered to be the limit of statistical significance for comparison purposes.

## Results

### Pre-implantation embryonic development was inhibited by LIF-siRNA

**Fig 1:**
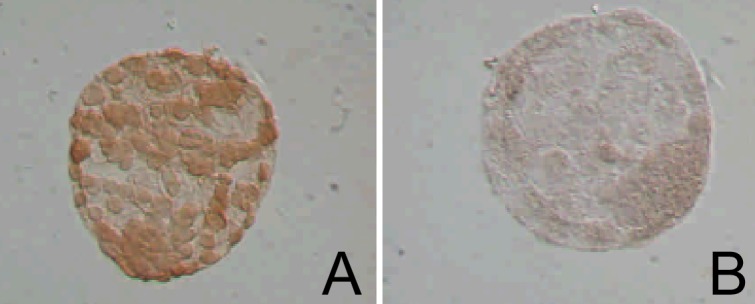
Immunocytochemical analysis of LIF protein expression on Days 5 in mouse embryos. (A) The blastocyst was from scrambled-siRNA treated group. (B) The blastocyst was from siRNA-LIF treated group. (200X).

LIF-siRNA was performed (Table 1) to confirm the data from the LIF-antisense oligonucleotide treatment in the previous experiment [16]. Our research showed that LIF-siRNA had significantly inhibited the embryonic development from the morula stage into the blastocyst stage at 41.7% development rate. Furthermore, a visual observation through immunocytochemical analysis of LIF protein expression of siRNA-treated embryos in the blastocyst stage revealed affirmative signs of lowered LIF performance (Fig 1). These results correlate with the effect of the LIF-antisense oligonucleotide treatment.

**Table 1:** Percentages (%) of mouse embryos developing into different pre-implantation stages after microinjection of LIF-siRNA or scrambled-siRNA at the two-pronuclear stage.

	Untreated (n = 51)	Solution (n = 30)	scramble (n = 87)		LIF-siRNA (n = 24)
Two-cell	90.2	93.3		93.1	91.7
Four-cell	85.7	86.7		92.0	75.0
Morula	83.4	83.3		89.7	58.3^a^
Blastocyst	83.4	80.0		86.2	41.7^a^

^a^P<0.05 compared with the untreated group.

### Gene expression profiles of LIF-siRNA-treated embryos

**Fig 2:**
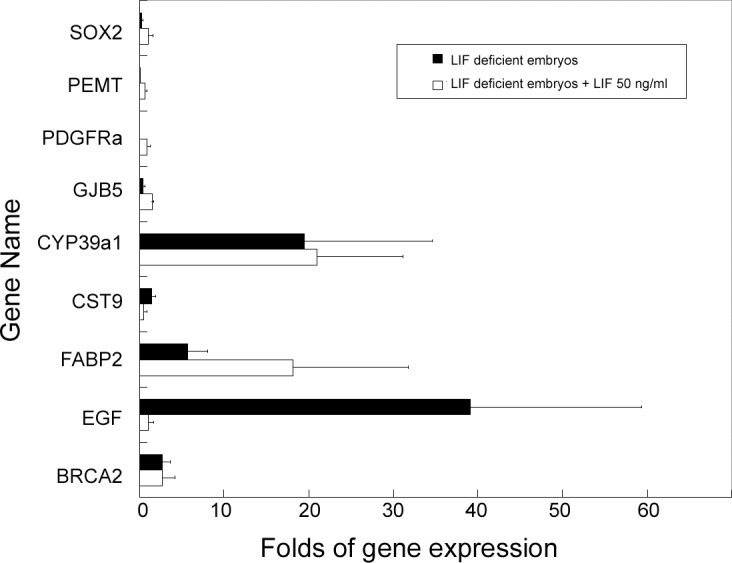
The change folds from quantification of gene expression on morula stage mouse embryos after microinjection of 1.5 fmol LIF-siRNA at mouse 2PN stage (N = 3) and supplemented with 50 ng/ml LIF protein, compared with the scrambled-siRNA treated embryos. The gene expressions were expressed as mean from 3 repeats and the bars are designed as standard deviations.

The microarray test comparing gene expression profiles of LIF-siRNA-treated and scrambled-siRNA-treated mouse embryos were performed twice for each group to ensure consistency of gene expression data. The microarray data revealed 216 down-expression (S2 Table) and 153 up-expression genes (S3 Table), of which, four down-regulated genes (SOX2, PEMT, PDGFRA, and GJB5) and five up-regulated genes (CYP39a1, CST9, FABP2, EGF, and BRCA2) (Table 2) were found to be significantly varied in expression at the morula stage in LIF-deficient embryos. Quantitative real-time RT-PCR performed using the primers described in Table 3 confirmed the microarray findings (Fig 2).

**Table 2:** Comparison different gene expression of mouse embryos in morula stage treated with oligonucleotide of LIF-siRNA and scrambled-siRNA at mouse 2PN stage by a statistical approach revealed up- and down regulated genes at morular stage.

N0.	Gene name	UniGene	Gene symbol	Function	Express^a^
1	SRY-box containing gene 2	Mm.65396	SOX2	Cell differentiation	DR (3)
2	Phosphatidylethanolamine N-methyltransferase	Mm.452195	PEMT	Metabolism	DR (4)
3	Gap junction membrane channel protein beta 5	Mm.26859	GJB5	Cell communication	DR(29)
4	Platelet derived growth factor receptor alpha	Mm.221403	PDGFRA	Cell communication	DR(166)
5	Cytochrome P450, 39a1	Mm.376968	CYP39a1	Metabolism	UR(1)
6	Cystatin 9	Mm.21005	CST9	Unknown	UR(4)
7	Fatty acid binding protein 2, intestinal	Mm.28398	FABP2	Cell physiology	UR(23)
8	Epidermal growth factor	Mm.252481	EGF	Metabolism	UR(31)
9	Breast cancer 2	Mm.236256	BRCA2	Unknown	UR(107)

^a^DR, down-regulation; UR, up-regulation. The number in the bracket is the series number of the two down-regulation and up-regulation groups in the S1 Table and S2 Table.

**Table 3:** Primer sequences of genes and Temperature (Tm) for annealing in real-time RT-RCR.

Gene symbol		Primer sequence	Length (bp)	Tm (°C)
SOX2	R	5’-ATGCACAACTCGGAGATCAG-3’	130	55
	F	5’-TATAATCCGGGTGCTCCTTC-3’		
PEMT	R	5’-GGCATCTGCATCCTGCTTTT-3’	70	55
	F	5’-TGGGCTGGCTCATCATAGC-3’		
GJB5	R	5’-AGTTCATGGTGGACTCAA-3’	110	55
	F	5’-GAATTCTGATGCTTG-3’		
PDGFRA	R	5’-GGAGAAGGTCTCAGGAGCTATG-3’	119	55
	F	5’-CGTTTGGGAGGATAGAGGGTAA-3’		
CYP39a1	R	5’-AGGTCATCTTCAGACACTTTA-3’	111	55
	F	5’-ACTTGGATATATCCTGTCTCA-3’		
CST9	R	5’-GAGTTCTGGCTCCATGTCCT-3’	105	55
	F	5’-GCCTTCGAGACAGGAGTGAT-3’		
FABP2	R	5’-GAGCAACGCTGAAGAGCTAA-3’	94	55
	F	5’-GCTGGAGACCAGTGCTGATA-3’		
EGF	R	5’-CTGGTCCTGCTGCTCCTCTT-3’	144	55
	F	5’-TCCGCTTGGCTCATCACA-3’		
BRCA2	R	5’-CTCTACCACTGGACCAGTCGC-3’	126	55
	F	5’-TCAGAGGCGGACTGACAACA-3’		

### Growth factor supplementation in LIF-deprived embryos

After supplying LIF-deprived embryos with 50 ng/ml LIF protein, a significantly higher percentage of blastocysts continued to develop compared with that in the LIF gene-impaired group without LIF supplement (P<0.05). Furthermore, in the LIF-deprived embryos given LIF supplement, EGF, CST9, GJB5, PDGFRA, PEMT, and SOX2 expression levels were restored in the morula stage, although CYP39al, FABP2, and BRCA2 expressions remained elevated after the LIF supplement (Fig 2). The data suggest that the expression of these genes may be closely affected by LIF.

Many reports indicated that the more commonly secreted growth factors from the reproductive tract are LIF, IGF-II, EGF, GM-CSF, and TGFβ [24]. Based on the reaction of EGF to LIF supplementation as mentioned above, we speculate that EGF assisted in the development of LIF-deficient embryo. Therefore, to observe the reaction of LIF and EGF on embryos, as well as that of the other growth factors (24), the embryos were injected with LIF-siRNA and supplemented with one of the growth factors (i.e., LIF, IGF-II, EGF, GM-CSF, and TGFβ) for each group in 50 ng/ml dosage. On the 5th day of the experiment, the results revealed that supplementing with LIF or EGF caused the rate of blastocyst development to recover significantly to 83% and 93% of the control group, respectively (Table 4). However, the other supplements did not produce significant recovery of development rate, thereby confirming the role of EGF assistance in the development of LIF-deficient embryo.

**Table 4:** Compared the embryo development rate from growth factors supplement in mouse embryos treated with LIF SiRNA.

Stage	Untreated (n = 62)	Scramble^a^ (n = 60)	LIF-siRNA (1.5 fmol)					
			Vehicle (*n* = 75)	EGF (50 ng/ml) (*n* = 66)	GM-CSF (50 ng/ml) (*n* = 72)	IGF II (50 ng/ml) (*n* = 75)	TGFβ (50 ng/ml) (n = 68)	LIF (50 ng/ml) (*n* = 77)
Two-cell	93.5	93.5	93.0	92.4	83.3	85.7	83.9	93.0
Four-cell	90.3	90.3	83.1	86.4	73.6	75.7	74.2	77.5
Morula	87.1	87.1	69.0	84.8	62.5	54.3	66.1	73.2
Blastocyst	82.3 ^b^	81.7 ^b^	43.6^a^	75.8^b^	58.3^a^	42.9^a^	48.4^a^	67.6 ^b^

^a^P<0.05 compared to scramble group

^b^P<0.05 compared to only LIF-siRNA treated group

Results of differential staining of blastocysts (Table 5) indicated that the diameter of blastocysts derived from embryos treated with LIF-siRNA was significantly decreased compared with that of the scrambled-siRNA-treated embryos. Moreover, these blastocysts also had significantly lower numbers of blastomeres and cells in ICM and TE. A significantly lower ICM:TE ratio was also found in these embryos (P<0.01). After supplementation with EGF and LIF at the 2PN stage, all of the parameters were improved, but IGF-II, GM-CSF, or TGFβ did not positively affect embryo stage progression.

**Table 5:** Changes in the number of cells in the inner cell mass (ICM) and trophectoderm (TE) of the blastocysts derived from murine embryos after microinjection of LIF SiRNA at the two-pronuclear stage.

	Untreated (n = 62)	Scramble (n = 60)	LIF-siRNA (1.5 fmol)					
			vehicle (n = 75)	EGF 50ng/ml (*n* = 66)	GM-CSF 50ng/ml (*n* = 72)	IGF II 50ng/ml (*n* = 75)	TGFβ 50ng/ml (n = 68)	LIF 50ng/ml (*n* = 77)
Blastulation rate (%)	82.3(51/62)	81.7(49/60)	43.6(33/75)	75.8(50/66)	58.3(42/72)	42.7(32/75)	48.5(33/68)	67.5(52/77)
No. of blastocysts measured	31	36	31	31	31	30	31	31
Diameter of blastocysts (μm)^c^	119.2± 14.0^a^^,^^c^	113.5 ± 11.6 ^c^	100.2 ± 4.3^b^	105.6± 6.0^b^^,^^c^	105.3 ± 6.4 ^b^^,^^c^	102.4 ± 6.0 ^b^	105.1± 7.1 ^b^^,^^c^	106.1 ± 5.8 ^b^^,^^c^
No. of blastomeres	63.5 ± 11.6 ^c^	64.4 ± 6.7 ^c^	40.4 ± 7.1^b^	44.4±8.0 ^b^^,^^c^	44.1±10.1^b^	48.1±8.7 ^b^^,^^c^	45.7±7.4 ^b^^,^^c^	46.1± 8.1 ^b^^,^^c^
No of cells in ICM	22.9 ± 3.5 ^c^	22.2 ± 2.7 ^c^	11.2 ± 2.4^b^	14.2 ±2.8 ^b^^,^^c^	12.1 ±3.7 ^b^	12.8±3.4 ^b^^,^^c^	13.2±2.5 ^b^^,^^c^	14.8 ± 2.6 ^b^^,^^c^
No of cells in TE	40.6± 8.7 ^c^	42.2± 5.7 ^c^	29.2± 5.3^b^	30.1 ±6.3^b^	32.0 ±7.6^b^	35.3 ±6.1 ^b^^,^^c^	32.4 ±6.4 ^b^^,^^c^	30.1± 6.3 ^b^
Ratio of ICM/TE cells (%)	58.7± 14.4 ^c^	53.4± 12.3 ^c^	38.5 ± 5.1^b^	48.5±10.6 ^b^^,^^c^	38.4 ±10.8 ^b^	36.4±8.6 ^b^	42.1±9.4^b^	50.3± 8.1 ^b^^,^^c^

^a^Mean ± Standard deviation.

^b^P <0.05 compared to the scramble group

^c^P<0.05 compared to only LIF-siRNA treated group

### Growth factor supplementation in EGF-deprived embryos

EGF-siRNA was employed to confirm the role of EGF, in which the embryonic development from the morula stage into the blastocyst stage was significantly inhibited at 59.4% development rate (Table 6). After supplying EGF-deprived embryos with 50 ng/ml LIF or EGF protein, a significantly higher percentage of blastocysts continued to develop. Moreover, when both LIF-siRNA and EGF-siRNA were employed, the embryonic development from the morula stage into the blastocyst stage was significantly inhibited at 18% development rate (Table 6). After supplying LIF- and EGF-deprived embryos with 50 ng/ml LIF or EGF protein, a significantly higher percentage of blastocysts also continued to develop, indicating that EGF and LIF can compensate for the deficiency of either of them during early embryonic development.

**Table 6:** Compared the embryo development rate from growth factors supplement in mouse embryos treated with EGF-siRNA and/or LIF-siRNA.

Stage	Untreated (n = 36)	EGF (*n* = 35)	LIF (*n* = 35)	Scramble (*n* = 42)	EGF-siRNA			LIF-siRNA			EGF-siRNA + LIF-siRNA		
					Vehicle (*n* = 34)	EGF (*n* = 30)	LIF (*n* = 34)	Vehicle (*n* = 36)	EGF (n = 34)	LIF (*n* = 32)	Vehicle (*n* = 40)	EGF (*n* = 36)	LIF (*n* = 36)
Two-cell	100.0	100.0	91.4	92.9	85.3	96.7	97.1	88.9	87.5	88.2	72.5	97.2	91.7
Four-cell	97.2	97.1	85.7	88.1	82.4	96.7	91.2	86.1	84.4	79.4	47.5 ^a^	80.6	80.6
Morula	94.4 ^d^	91.4 ^d^	85.7 ^d^	88.1 ^d^	70.6 ^d^	90.0 ^d^	88.2 ^d^	77.8 ^d^	78.1 ^d^	70.6 ^d^	25.0 ^a^^,^^b^^,^^c^	61.1 ^d^	55.6
Blastocyst	80.6^d^	88.6 ^d^	77.1 ^d^	81.0 ^d^	55.9 ^d^	76.7 ^d^	76.5 ^d^	33.3 ^a^	50.0 ^d^	50.0 ^d^	17.5 ^a^^,^^b^	47.2 ^d^	47.2 ^d^

^a^P<0.05 compared to blank group

^b^P<0.05 compared to only EGF-siRNA treated group

^c^P<0.05 compared to only LIF-siRNA treated group

^d^P<0.05 compared to EGF-siRNA and LIF-siRNA treated group

### JAK/STAT3 pathway in LIF and EGF signaling

LIF binds to LIFR and can react with glycoprotein 130 (gp130), which are both situated on a receptor complex [19]. The complex is known to activate the JAK/STAT3, MAPK, and PI3K signaling pathways. To determine the mechanism of LIF in embryonic development, embryos were treated with combinations of LIF antisense oligonucleotide and/or with several kinase inhibitors, including JAK inhibitor JAK I-II, MEK inhibitor PD98059, and PI3K inhibitor LY294002 (Table 7). Combination treatment with LIF antisense oligonucleotide and any one inhibitor all largely inhibited the blastocyst formation. However, similar to the above results, when the LIF antisense oligonucleotide-treated embryo were co-treated with LIF or EGF, the inhibition was recovered significantly from 52% of control to 83% and 93%, respectively. When co-treatment with LIF or EGF was added to the combination treatments, development recovery was achieved from 25.4% of control to 73.7% and 46.4%, respectively, under MEK signaling pathway inhibition, and from 31.3% of control to 67.7% and 65.8%, respectively, under PI3K signaling pathway inhibition; however, under inhibition of the JAK/STAT3 signaling pathway, the embryonic development was only recovered from 5.3% of control to 25.9% and 11.8%, respectively, indicating the importance of the JAK/STAT3 signaling pathway in the LIF or EGF supplementation. Furthermore, JAK/STAT3 signaling could be a part of the pathway that transmits LIF and EGF signals [25]. Thus, the EGFR inhibitor PD168393 was used to determine whether their signals are also a partly mediated through the EGFR/STAT3 pathway. The results showed that the rate of the blastocyst formation was also significantly inhibited by the EGFR inhibitor. Combination treatment with LIF antisense oligonucleotide and the EGFR inhibitor synergistically inhibited the blastocyst formation, and with co-treatment with LIF or EGF, the blastocyst development rate was altered from 25.4% of control to 31.8% and 20.6%, respectively. These results suggest that the effects of LIF and EGF can be partly mediated by the EGFR/STAT3 signaling pathway.

**Table 7:** Compared the blastocyst development rate from growth factors (50 ng/ml) supplement in mouse embryos treated with LIF antisense and/or kinase inhibitors.

Imhibitor	Untreated	EGF	LIF	NaCl	Scramble	Inhibitor	LIF antisense					
							vehicle	EGF	LIF	Inhibitor	Inhibitorand EGF	Inhibitor and LIF
MEK PD98059 (100 μM)	73.9 ^b^^,^^c^(n = 46)	71.9 ^b^^,^^c^(*n* = 32)	80.0 ^b^^,^^c^(*n* = 35)	75.0 ^b^^,^^c^(*n* = 40)	71.1 ^b^^,^^c^(*n* = 38)	40.5(*n* = 42)	28.9^a^(*n* = 45)	76.9^b^^,^^c^(n = 39)	67.5^b^^,^^c^(*n* = 40)	18.8^a^(*n* = 32)	54.5 ^c^(n = 44)	34.3(*n* = 35)
PI3K LY294002 (20 μM)	73.9 ^b^(n = 46)	80.0 ^b^(*n* = 30)	81.3 ^b^(*n* = 32)	75.0 ^b^(*n* = 40)	69.4 ^b^(*n* = 36)	42.5(*n* = 40)	28.9^a^(*n* = 45)	76.9^b^(n = 39)	67.5^b^(*n* = 40)	23.1^a^(*n* = 39)	50.0(n = 44)	48.6(*n* = 35)
JAKJAK I-II (0.1 μM)	85.0^b^^,^^c^(n = 40)	87.5 ^b^^,^^c^(*n* = 40)	90.0 ^b^^,^^c^(*n* = 40)	75.0 ^b^^,^^c^(*n* = 40)	67.5 ^b^^,^^c^(*n* = 40)	13.3^a^(*n* = 45)	27.5^a^^,^^c^(*n* = 40)	86.8^b^^,^^c^(n = 38)	75.0^b^^,^^c^(*n* = 40)	4.5^a^^,^^b^(*n* = 44)	22.0^a^^,^^c^(n = 41)	10.6^a^(*n* = 47)
EGFR PD168393 (2 μM)	73.9 ^b^^,^^c^(n = 46)	75.0^b^^,^^c^(*n* = 40)	84.2^b^^,^^c^(*n* = 38)	65.0^b^^,^^c^(*n* = 40)	68.9^b^^,^^c^(*n* = 41)	20.0^a^(*n* = 32)	28.9^a^(*n* = 45)	84.2^b^^,^^c^(n = 38)	75.0^b^^,^^c^(*n* = 40)	18.8 ^a^(*n* = 32)	23.5 ^a^(n = 34)	15.2 ^a^(*n* = 33)

^a^P<0.05 compared to blank group

^b^P<0.05 compared to only LIF-antisense treated group

^c^P<0.05 compared to LIF-antisense and inhibitor treated group

## Discussion

### Either LIF or EGF is needed during development of pre-implantation embryo

A previous study [16] and the present data confirmed that LIF deficiency can significantly decrease the development of embryos into blastocysts, as well as lower the number of ICM and TE. Moreover, the cDNA microarray data and the growth factor supplementation experiments showed that the elevated EGF level in LIF-deprived embryos can compensate for LIF-deficiency, suggesting that EGF holds similar functions and mechanisms as LIF during pre-implantation embryonic development. This finding is consistent with reports of EGF promoting nuclear and cytoplasmic maturation of human, bovine, and porcine oocytes and enhancing the development of embryos from 2-cell stage to blastocyst [26–29]. Dadi et al. [30] also stated the importance of EGF to the blastocyst development; they reported that the reduction of EGF and EGF receptor expression decreased the expression of maternally inherited and embryonically expressed genes and reduced the total number of differentiated cells in blastocysts in pre-implantation mouse embryos. Furthermore, although many conflicting results are obtained, the differences in the effects of LIF or EGF treatment during blastocyst formation may be attributed to the environment of the embryos (e.g., stress or deprivation of essential developmental conditions) [31, 32]. Such difference may explain the results of our present study on LIF and EGF gene-deprived embryos, and thus, our results suggest that sufficient amount of one of the two growth factors is needed during pre-implantation embryonic development.

### JAK2/STAT3 pathway maintains the effects of LIF and EGF

Investigation of the signaling pathways showed that, of the three pathways that are activated through LIF receptors containing the gp130 subunit [25], all significantly halted/slowed embryonic development when inhibited individually; however, the restoration properties of EGF and LIF supplementation/treatment became ineffective only when the JAK2/STAT3 pathway was closed by an inhibitor. Thus, the JAK2/STAT3 pathway is important in LIF-derived embryogenesis. Other reports on the LIF-JAK-STAT3 signaling pathway and sustenance of self-renewal in mouse embryonic stem cells [21–22] and on STAT3-deficient mouse embryos and rapid degeneration during embryogenesis [20] also indicated the significance of the STAT3 pathway. The relationship between STAT3 and EGF is originally found in mouse liver [33], and is usually observed in cancer/tumorigenesis [34–36]. This paper is the first to report their relationship in embryonic development. Perkins et al. [37] reported that EGF exerts its anti-apoptotic effect during the first-trimester trophoblast development independent of PI3K/Akt; however, the underlying mechanism was not reported. Our findings suggest that the anti-apoptotic effect could be due to the activation of the JAK2/STAT3 pathway, which maintained the effects of LIF and EGF.

### Effects of LIF and EGF are regulated by EGFR

Many reports indicate that EGFR is involved in pre-implantation embryonic development [38, 30], but the mechanism remains unclear. Viti et al. [25] reported that EGFRs elevate STAT3 expression and increase its phosphorylation by LIF during astrocyte development in the early embryonic cortex, suggesting that EGFRs regulate LIF responsiveness downstream of STAT3. The relationship between LIF- and EGFR-induced STAT3 activation is also found in various types of cells, such as lung carcinoma cells [39] and ovarian cancer cell lines [35].We applied an EGFR inhibitor to the embryo before LIF and EGF supplementation to determine the role of EGFR in the LIF-STAT3 and EGF-STAT3 pathways during pre-implantation embryonic development. Our results showed that the recovery effects of both EGF and LIF in the LIF-deprived embryos can be inhibited by an EGFR inhibitor. This paper is the first to report that the effects of EGF and LIF can be regulated by EGFR during pre-implantation embryonic development, thereby providing a step into uncovering this particular mechanism in embryonic development. Further inquiry may focus on the modulation of STAT3 phosphorylation by LIF and EGF.

### EGF may be a compensatory factor during the development of LIF-deficient embryo

Based on our results, we propose that embryos of knockout mouse [7, 40] shown to develop to the blastocyst stage in vivo in the absence of LIF may have been rescued by other factors, such as EGF, which is produced from reproductive tract and can compensate for the lack of LIF. In embryonic stem cell self-renewal, one of the other candidate factors besides LIF is fibroblast growth factor 2 (FGF2), which acts via the fibroblast growth factor receptor (FGFR) pathway [21]. Modulation of FGF signaling in FGFR1- and FGFR2-null or FGF2 mutant mice alters blastocyst development and differentiation. FGF2 could maintain a function similar to LIF in mouse embryonic stem cells because FGF2 maintains embryonic stem cell self-renewal by supporting stable expression of extracellular matrix through activation of the PI3K/PKB pathway. Therefore, we suggest that EGF may also be a candidate factor during the development of LIF-deficient embryo. In addition, EGF may function through STAT3 to improve embryonic development. Further investigation is needed to determine the compensatory mechanism during pre-implantation.

## Supporting Information

S1 Table The changes of blastocyst development rate in the supplement with the different dosages of the kinase inhibitors [MEK inhibitor (PD98059), PI3K inhibitor (LY294002), JAK inhibitor (JAK I-II), and EGFR inhibitor (PD168393)]. (PDF)

S2 Table The down-expression genes of LIF-siRNA treated mouse embryos at the morula stage in microarray test. The ratio is the normalized expression ratio (by rank consistent lowness) of comparing gene expression profiles of 2.0-fmol siRNA treated embryos to scrambled siRNA treated embryos. The analysis was performed twice for each group in order to ensure consistency of gene expression data.
(PDF)

S3 Table The up-expression genes of LIF-siRNA treated mouse embryos at the morula stage in microarray test. The ratio is the normalized expression ratio (by rank consistent lowness) of comparing gene expression profiles of 2.0-fmol siRNA treated embryos to scrambled siRNA treated embryos. The analysis was performed twice for each group in order to ensure consistency of gene expression data.
(PDF)
