# Supplementary material for: Requirement of Leukemia Inhibitory Factor or Epidermal Growth Factor for Pre-Implantation Embryogenesis via JAK/STAT3 Signaling Pathways
Source: PLoS One. 2016 Apr 20;11(4):e0153086. doi: 10.1371/journal.pone.0153086 (PMC4838257; doi:10.1371/journal.pone.0153086)
Supplement: S1 Table — (PDF) [file pone.0153086.s001.pdf]

S1 Table. The changes of blastocyst development rate in the supplement with the different dosages of the kinase inhibitors [MEK inhibitor (PD98059), PI3K inhibitor (LY294002), JAK inhibitor (JAK I-II), and EGFR inhibitor (PD168393)].

Imhibitor	Untreated (n=46)	PD98059 (μM)					LY294002 (μM)					JAK I-II (μM)				PD168393 (μM)				
		500	100	50	10	0.1	100	50	20	10	1	1	0.1	0.01	0.001	10	2	1	0.1	0.01
Two-cell	98.2	70.4	88.0	83.3	92.9	90.0	10.0[a]	77.5	100.0	85.7	95.5	87.5	85.1	81.5	89.2	41.2[a]	90.9	84.0	80.0	100.0
Four-cell	91.2	18.5[a]	76.0	75.0	85.7	90.0	10.0[a]	60.0[a]	81.5	75.0	90.9	68.8	70.2	55.6[a]	64.9[a]	29.4[a]	84.8	72.0	73.3	78.6
Morula	91.2	14.8[a]	64.0[a]	75.0	78.6	90.0	6.7[a]	27.5[a]	51.9[a]	64.3[a]	77.3	62.5[a]	51.1[a]	51.9[a]	59.5[a]	11.8[a]	60.6[a]	68.0[a]	73.3	78.6
Blastocyst	84.2	14.8[a]	60.0[a]	66.7[a]	78.6	80.0	3.3[a]	17.5[a]	51.9[a]	64.3[a]	72.7	18.8[a]	48.9[a]	51.9[a]	56.8[a]	11.8[a]	51.5[a]	52.0[a]	73.3	78.6

[a]P<0.05 compared to blank group
