# Supplementary material for: Requirement of Leukemia Inhibitory Factor or Epidermal Growth Factor for Pre-Implantation Embryogenesis via JAK/STAT3 Signaling Pathways
Source: PLoS One. 2016 Apr 20;11(4):e0153086. doi: 10.1371/journal.pone.0153086 (PMC4838257; doi:10.1371/journal.pone.0153086)
Supplement: S2 Table — The ratio is the normalized expression ratio (by rank consistent lowness) of comparing gene expression profiles of 2.0-fmol siRNA treated embryos to scrambled siRNA treated embryos. The analysis was performed twice for each group in order to ensure consistency of gene expression data. (PDF) [file pone.0153086.s002.pdf]

S2 Table. The down-expression genes of LIF-siRNA treated mouse embryos at the morula stage in microarray test. The ratio is the normalized expression ratio (by rank consistent lowness) of comparing gene expression profiles of 2.0-fmol siRNA treated embryos to scrambled siRNA treated embryos. The analysis was performed twice for each group in order to ensure consistency of gene expression data.

No.	UniGene	GeneName	Ratio
1	Mm.29296	RIKEN cDNA 1110003H02 gene	0.10
2	Mm.202383	hepatic nuclear factor 4	0.12
3	Mm.65396	SRY-box containing gene 2	0.13
4	Mm.2731	phosphatidylethanolamine N-methyltransferase	0.14
5	Mm.276116	ankyrin-like repeat protein	0.14
6	Mm.400747	Eph receptor A4	0.15
7	Mm.288381	fibulin 5	0.15
8	Mm.273379	RIKEN cDNA 1810032P22 gene	0.15
9	Mm.25802	ESTs	0.17
10	Mm.1894	CD1d1 antigen	0.18
11	Mm.224233	RIKEN cDNA 2810407E23 gene	0.18
12	Mm.4462	SH3-domain binding protein 1	0.19
13	Mm.29210	aplysia ras-related homolog N (RhoN)	0.20
14	Mm.4512	RIKEN cDNA 1110001J05 gene	0.20
15	Mm.43636	zinc finger protein 289	0.20
16	Mm.245724	RIKEN cDNA 0610011L04 gene	0.21
17	Mm.35754	cytosolic sorting protein Phosphofurin acidic cluster sorting protein	0.21
18	Mm.259795	Ras homolog gene family, member G (RhoG)	0.21
19	Mm.252316	glutathione synthetase	0.22
20	Mm.76286	ESTs	0.22
21	Mm.33765	ADP-ribosylation factor 1 GTPase activating protein	0.22
22	Mm.441439	E74-like factor 3	0.22
23	Mm.33801	ESTs	0.22
24	Mm.1775	hematological and neurological expressed sequence 1	0.22
25	Mm.41868	RIKEN cDNA 2810405K02 gene	0.23
26	Mm.197829	Tu translation elongation factor, mitochondrial	0.23
27	Mm.149	RAD52 homolog, (S. cerevisiae)	0.24
28	Mm.26859	gap junction membrane channel protein beta 5	0.24
29	Mm.394	intercellular adhesion molecule 2	0.25
30	Mm.687	aplysia ras-related homolog B (RhoB)	0.25

31	Mm.179349	RIKEN cDNA 2700050C19 gene	0.25
32	Mm.30907	ESTs	0.25
33	Mm.1971	calreticulin	0.25
34	Mm.3991	solute carrier family 25 (mitochondrial carrier; adenine nucleotide translocator), member 10	0.25
35	Mm.24714	ESTs	0.26
36	Mm.121859	RIKEN cDNA 2310020F24 gene	0.26
37	Mm.381194	RIKEN cDNA 2310011F05 gene	0.26
38	Mm.205830	guanosine diphosphate (GDP) dissociation ihibitor 1	0.27
39	Mm.63549	ESTs	0.27
40	Mm.26995	thimet oligopeptidase 1	0.27
41	Mm.39700	ESTs	0.28
42	Mm.104955	serine protease inhibitor, Kunitz type 1	0.28
43	Mm.143768	f-box only protein 3	0.28
44	Mm.982	endothelial differentiation sphingolipid G-protein-coupled receptor 1	0.30
45	Mm.247956	RIKEN cDNA 1200011D11 gene	0.30
46	Mm.344648	CD97 antigen	0.30
47	Mm.24173	ESTs	0.30
48	Mm.1583	lymphocyte antigen 6 complex, locus C	0.30
49	Mm.358616	keratin complex 2, basic, gene 8	0.30
50	Mm.45294	ESTs	0.30
51	Mm.298798	cyclin-dependent kinase 5	0.30
52	Mm.8180	lymphocyte antigen 6 complex	0.31
53	Mm.272675	solute carrier family 20, member 1	0.31
54	Mm.41864	RIKEN cDNA 1190005K07 gene	0.31
55	Mm.29096	nuclear receptor binding factor 1	0.31
56	Mm.24069	feminization 1 a homolog (C. elegans)	0.31
57	Mm.71925	ESTs	0.32
58	Mm.42948	thioredoxin peroxidase, pseudogene 1	0.32
59	Mm.196376	DNA segment, Chr 8, ERATO Doi 580, expressed	0.32
60	Mm.21912	RIKEN cDNA 2810425J22 gene	0.32
61	Mm.29778	RIKEN cDNA 3230402M22 gene	0.32
62	Mm.6700	eukaryotic translation initiation factor 4E binding protein 1	0.33
63	Mm.23314	ESTs	0.33
64	Mm.190650	N-acetylglucosamine kinase	0.33
65	Mm.213023	actin, alpha 2, smooth muscle, aorta	0.33
66	Mm.285906	suppressor of Ty 5 homolog (S. cerevisiae)	0.33

67	Mm.358698	RIKEN cDNA 5330403M05 gene	0.33
68	Mm.86746	ESTs	0.34
69	Mm.292865	actin, gamma 2, smooth muscle, enteric	0.34
70	Mm.42196	nuclear protein 95	0.34
71	Mm.4259	tumor-associated calcium signal transducer 1	0.34
72	Mm.347009	peroxiredoxin 2	0.34
73	Mm.24606	ESTs	0.34
74	Mm.686	actin, alpha, cardiac muscle	0.35
75	Mm.24662	S100 calcium binding protein A1	0.35
76	Mm.469099	proline-serine-threonine phosphatase-interacting protein 1	0.35
77	Mm.257765	chloride intracellular channel 4 (mitochondrial)	0.35
78	Mm.26743	apolipoprotein A-I	0.35
79	Mm.441542	glutathione S-transferase, pi 2	0.35
80	Mm.55181	ESTs	0.35
81	Mm.34098	ESTs	0.35
82	Mm.28386	RIKEN cDNA 3110001D19 gene	0.35
83	Mm.24255	transducin-like enhancer of split 3, homolog of Drosophila E(spl)	0.35
84	Mm.31914	ESTs	0.35
85	Mm.270681	heat shock protein, 110 kDa	0.35
86	Mm.41928	ESTs	0.35
87	Mm.222329	calcium/calmodulin-dependent protein kinase IV	0.35
88	Mm.192991	metallothionein 1	0.35
89	Mm.28130	PCTAIRE-motif protein kinase 3	0.35
90	Mm.258633	stress-induced phosphoprotein 1	0.36
91	Mm.200392	ESTs	0.36
92	Mm.329655	cofilin 1, non-muscle	0.36
93	Mm.24506	ESTs	0.36
94	Mm.268383	ribosomal protein S6 kinase, 90kD, polypeptide 2	0.36
95	Mm.130	cytokine inducible SH2-containing protein 1	0.36
96	Mm.44197	RIKEN cDNA 2410012F02 gene	0.36
97	Mm.21868	raf-related oncogene	0.36
98	Mm.30016	Mus musculus Gdi-1 mRNA for RhoGDI-1, complete cds	0.36
99	Mm.103351	ATP-binding cassette, sub-family A (ABC1), member 7	0.36
100	Mm.23851	ESTs	0.37
101	Mm.263185	vacuolar protein sorting 45 (yeast)	0.37
102	Mm.330057	RIKEN cDNA 2900002K07 gene	0.37
103	Mm.4973	zinc finger protein 147	0.37

104	Mm.2409	alcohol dehydrogenase 1	0.37
105	Mm.36078	ESTs	0.37
106	Mm.9287	microtubule associated testis specific serine/threonine protein kinase	0.37
107	Mm.440086	glutathione S-transferase, mu 2	0.37
108	Mm.196289	DNA segment, Chr 15, Wayne State University 122, expressed	0.38
109	Mm.38060	ESTs	0.38
110	Mm.38087	ESTs	0.38
111	Mm.213873	integrin beta 4	0.38
112	Mm.22240	expressed sequence AU022188	0.39
113	Mm.29034	ESTs, Moderately similar to unknown [H.sapiens]	0.39
114	Mm.41388	ESTs	0.39
115	Mm.247946	RIKEN cDNA 2310002A12 gene	0.39
116	Mm.7244	anterior gradient 2 (Xenepus laevis)	0.39
117	Mm.142524	ESTs	0.39
118	Mm.34404	sema domain, immunoglobulin domain (Ig), transmembrane domain (TM) and short cytoplasmic domain, (semaphorin) 4G	0.39
119	Mm.234502	myosin Ic	0.39
120	Mm.329287	GATA-binding protein 6	0.39
121	Mm.125465	RIKEN cDNA 3110031B13 gene	0.40
122	Mm.2055	matrix metalloproteinase 12	0.40
123	Mm.481405	tumor rejection antigen gp96	0.40
124	Mm.331547	aconitase 1	0.40
125	Mm.141758	hemoglobin X, alpha-like embryonic chain in Hba complex	0.40
126	Mm.22123	DNA segment, Chr 15, Wayne State University 75, expressed	0.40
127	Mm.390239	activin receptor IIB	0.41
128	Mm.4551	villin 2	0.41
129	Mm.1249	laminin, gamma 1	0.41
130	Mm.39061	adenylosuccinate synthetase 2, non muscle	0.41
131	Mm.10	spermidine synthase	0.41
132	Mm.262252	small EDRK-rich factor 2	0.41
133	Mm.157778	RIKEN cDNA 2610034E13 gene	0.41
134	Mm.274846	integrin linked kinase	0.41
135	Mm.17911	lectin, galactose binding, soluble 6	0.41
136	Mm.38492	ESTs, Weakly similar to FOR4 MOUSE FORMIN 4 [M.musculus]	0.41
137	Mm.6994	hydroxyacyl-Coenzyme A dehydrogenase, type II	0.41
138	Mm.341434	lectin, galactose binding, soluble 9	0.41

139	Mm.12393	ESTs	0.42
140	Mm.28426	ESTs, Highly similar to histone acetyltransferase [H.sapiens]	0.42
141	Mm.43345	ESTs	0.42
142	Mm.317331	Yamaguchi sarcoma viral (v-yes-1) oncogene homolog	0.42
143	Mm.424974	MARCKS-like protein	0.42
144	Mm.334144	SWAP complex protein, 70 kDa	0.42
145	Mm.2923	interleukin 2 receptor, gamma chain	0.42
146	Mm.290442	polycystic kidney disease 1 homolog	0.42
147	Mm.2230	dead ringer homolog 1 (Drosophila)	0.42
148	Mm.273538	tubulin, beta 5	0.42
149	Mm.22594	ESTs, Moderately similar to ZINC FINGER PROTEIN 32 [Homo sapiens]	0.43
150	Mm.88645	testis derived transcript	0.43
151	Mm.39061	adenylosuccinate synthetase 2, non muscle	0.43
152	Mm.3179	immunoglobulin mu binding protein 2	0.43
153	Mm.214950	actin, alpha 1, skeletal muscle	0.43
154	Mm.291235	diacylglycerol kinase, alpha (80 kDa)	0.43
155	Mm.33253	DNA segment, Chr 2, Brigham & Women's Genetics 0886 expressed	0.43
156	Mm.30155	ATPase, H+ transporting, lysosomal (vacuolar proton pump) 16kD	0.43
157	Mm.44225	ribosomal protein, mitochondrial, L26	0.44
158	Mm.33584	ESTs, Weakly similar to C46027 neurotransmitter transporter - mouse [M.musculus]	0.44
159	Mm.27406	ESTs	0.44
160	Mm.141587	DNA-binding protein A	0.45
161	Mm.19182	hepsin	0.45
162	Mm.244975	breast cancer 1	0.45
163	Mm.196627	core promoter element binding protein	0.45
164	Mm.221403	platelet derived growth factor receptor, alpha polypeptide	0.45
165	Mm.5220	histone gene complex 2	0.45
166	Mm.41272	Mus musculus adult male liver cDNA, RIKEN full-length enriched library, clone:1300016P12, full insert sequence	0.45
167	Mm.4651	kinesin-associated protein 3	0.45
168	Mm.38993	calsyntenin 1	0.45
169	Mm.29320	ESTs	0.45
170	Mm.379002	ribosomal protein L28	0.46
171	Mm.177650	RIKEN cDNA 1300003D18 gene	0.46
172	Mm.141864	FK506 binding protein 8 (38 kDa)	0.46

173	Mm.589	glucose phosphate isomerase 1 complex	0.46
174	Mm.36417	ESTs, Weakly similar to unnamed protein product [H.sapiens]	0.46
175	Mm.8142	faciogenital dysplasia homolog	0.46
176	Mm.290868	target of myb1 homolog (chicken)	0.46
177	Mm.156	downstream of tyrosine kinase 1	0.46
178	Mm.358633	splicing factor 3a, subunit 2, 66kD	0.46
179	Mm.12758	FK506 binding protein 4 (59 kDa)	0.46
180	Mm.3544	calcium channel beta 3 subunit	0.47
181	Mm.29728	DNA segment, Chr 13, Wayne State University 156, expressed	0.47
182	Mm.386752	ancient ubiquitous protein	0.47
183	Mm.87586	ESTs, Moderately similar to DRPLA [H.sapiens]	0.47
184	Mm.30456	ESTs, Weakly similar to T29012 hypothetical protein ZK328.7 - Caenorhabditis elegans [C.elegans]	0.47
185	Mm.1129	repeat family 3 gene	0.47
186	Mm.266972	double C2, gamma	0.47
187	Mm.31552	ESTs, Weakly similar to I49441 Mouse 19.5 mRNA, complete cds - mouse [M.musculus]	0.48
188	Mm.34283	ESTs	0.48
189	Mm.22895	ESTs	0.48
190	Mm.28896	phosphatidylcholine transfer protein-like	0.48
191	Mm.236443	fatty acid synthase	0.48
192	Mm.7271	nuclear RNA export factor 1 homolog (S. cerevisiae)	0.48
193	Mm.439922	RIKEN cDNA 0610031J06 gene	0.48
194	Mm.271711	transgelin 2	0.48
195	Mm.265347	annexin A6	0.48
196	Mm.19726	dynein, axon, heavy chain 11	0.48
197	Mm.12917	multiple endocrine neoplasia 1	0.48
198	Mm.356689	acyl-Coenzyme A oxidase	0.49
199	Mm.271809	Fas death domain-associated protein	0.49
200	Mm.27291	cyclin D3	0.49
201	Mm.4222	triosephosphate isomerase	0.49
202	Mm.27921	ESTs, Highly similar to GAK RAT CYCLIN G-ASSOCIATED KINASE [R.norvegicus]	0.49
203	Mm.23298	ESTs	0.49
204	Mm.22067	transmembrane domain protein regulated in adipocytes 40 kDa	0.49
205	Mm.3130	kinesin heavy chain member 4	0.49
206	Mm.46176	RIKEN cDNA 1500041B02 gene	0.49
207	Mm.236868	ribosomal protein S3	0.49

208	Mm.31942	ESTs, Highly similar to hypothetical protein [M.musculus]	0.50
209	Mm.306256	heat shock protein, 84 kDa 1	0.50
210	Mm.1081	ATPase, H+ transporting, lysosomal (vacuolar proton pump), 42 kDa	0.50
211	Mm.277661	low density lipoprotein receptor-related protein associated protein 1	0.50
212	Mm.29133	budding uninhibited by benzimidazoles 1 homolog, beta (S. cerevisiae)	0.50
213	Mm.371592	ubiquitin B	0.50
214	Mm.22228	gene rich cluster, C8 gene	0.50
215	Mm.30262	Kruppel-like factor 5	0.50
216	Mm.31858	ESTs	0.50
