# Supplementary material for: Requirement of Leukemia Inhibitory Factor or Epidermal Growth Factor for Pre-Implantation Embryogenesis via JAK/STAT3 Signaling Pathways
Source: PLoS One. 2016 Apr 20;11(4):e0153086. doi: 10.1371/journal.pone.0153086 (PMC4838257; doi:10.1371/journal.pone.0153086)
Supplement: S3 Table — The ratio is the normalized expression ratio (by rank consistent lowness) of comparing gene expression profiles of 2.0-fmol siRNA treated embryos to scrambled siRNA treated embryos. The analysis was performed twice for each group in order to ensure consistency of gene expression data. (PDF) [file pone.0153086.s003.pdf]

S3 Table. The up-expression genes of LIF-siRNA treated mouse embryos at the morula stage in microarray test. The ratio is the normalized expression ratio (by rank consistent lowness) of comparing gene expression profiles of 2.0-fmol siRNA treated embryos to scrambled siRNA treated embryos. The analysis was performed twice for each group in order to ensure consistency of gene expression data.

No.	UniGene	GeneName	Ratio
1	Mm.376968	cytochrome P450, 39a1 (oxysterol 7alpha-hydroxylase)	14.5
2	Mm. 347441	forkhead box D1	14.3
3	Mm.33605	ESTs, Weakly similar to SKIP_HUMAN NUCLEAR PROTEIN SKIP [H.sapiens]	13.1
4	Mm.21005	cystatin 9	11.7
5	Mm.24511	ESTs	9.4
6	Mm.203916	3'-phosphoadenosine 5'-phosphosulfate synthase 2	9.1
7	Mm.33341	ESTs, Weakly similar to JE0343 terf protein - rat [R.norvegicus]	8.7
8	Mm.41182	ESTs	7.3
9	Mm.69031	ESTs, Moderately similar to unknown [M.musculus]	6.9
10	Mm.63584	RIKEN cDNA 1110030H18 gene	6.7
11	Mm.32744	opsin (encephalopsin)	6.5
12	Mm.327311	Mus musculus clone L5 uniform group of 2-cell-stage gene family mRNA, complete cds	6.4
13	Mm.38119	ESTs	6.4
14	Mm.327311	Mus musculus clone L3 variable group of 2-cell-stage gene family mRNA, complete cds	6.1
15	Mm.5	homeo box A10	6.1
16	Mm.391146	ESTs, Weakly similar to RST [M.musculus]	5.9
17	Mm.30872	ESTs	5.9
18	Mm.28010	ESTs, Weakly similar to RO52 MOUSE 52 KD RO PROTEIN [M.musculus]	5.8
19	Mm.31010	ESTs	5.7
20	Mm.1363	thyrotropin releasing hormone	5.6
21	Mm.348782	RIKEN cDNA 2410025L10 gene	5.5
22	Mm.87505	ESTs	5.4
23	Mm.28398	fatty acid binding protein 2, intestinal	5.4
24	Mm.24818	ESTs	5.3
25	Mm.896	interleukin 1 receptor, type I	5.2
26	Mm.96743	ESTs	5.0
27	Mm.104492	Mus musculus endogenous retroviral sequence MuERV-L gag, pol and dUTPase genes	5.0

28	Mm.466870	DNA segment, Chr 13, ERATO Doi 608, expressed	5.0
29	Mm.290447	RIKEN cDNA 2410049M19 gene	4.9
30	Mm.24749	ESTs	4.8
31	Mm.8534	epidermal growth factor	4.7
32	Mm.66283	ESTs	4.6
33	Mm.232525	RIKEN cDNA 2310008J16 gene	4.4
34	Mm.26700	ESTs, Highly similar to unnamed protein product [H.sapiens]	4.3
35	Mm.263514	RIKEN cDNA 4930569O04 gene	4.2
36	Mm.108054	DNA segment, Chr 1, Pasteur Institute 1	4.2
37	Mm.148342	deiodinase, iodothyronine, type I	4.2
38	Mm.14775	Mus musculus 10 days embryo cDNA, RIKEN full-length enriched library, clone:2610027B07, full insert sequence	4.1
39	Mm.300318	solute carrier family 6 (neurotransmitter transporter, serotonin), member 4	4.1
40	Mm.20461	gamma-glutamyl hydrolase	4.1
41	Mm.3243	protein C receptor, endothelial	4.1
42	Mm.290995	collapsin response mediator protein 1	4.0
43	Mm.3092	inhibin beta-B	4.0
44	Mm.167882	ESTs, Highly similar to DCC MOUSE TUMOR SUPPRESSOR PROTEIN DCC PRECURSOR [M.musculus]	3.9
45	Mm.158143	nuclear receptor subfamily 2, group F, member 2	3.8
46	Mm.21657	ESTs, Highly similar to ENDOTHELIN-CONVERTING ENZYME 1 E-1) [Rattus norvegicus]	3.8
47	Mm.31007	ESTs	3.8
48	Mm.23782	expressed sequence AI427652	3.8
49	Mm.297825	ATP-binding cassette, sub-family B (MDR/TAP), member 4	3.7
50	Mm.38342	RIKEN cDNA 2310005D12 gene	3.7
51	Mm.25249	ESTs	3.7
52	Mm.27243	DNA segment, Chr 9, ERATO Doi 85, expressed	3.6
53	Mm. 207496	protein kinase C, beta	3.6
54	Mm.216321	arginine-tRNA-protein transferase 1	3.6
55	Mm.7043	Ia-associated invariant chain	3.5
56	Mm.31005	ESTs	3.4
57	Mm.89048	coagulation factor II	3.4
58	Mm.31053	ESTs	3.3
59	Mm.28559	RIKEN cDNA 4632404H22 gene	3.3
60	Mm.272115	myomesin 2	3.3
61	Mm.34385	ESTs	3.2

62	Mm.371577	phosphodiesterase 8A	3.2
63	Mm.34221	ESTs, Moderately similar to S29319 transcription factor IIE-alpha [H.sapiens]	3.2
64	Mm.3865	seb4 mRNA	3.1
65	Mm.283281	RIKEN cDNA 9130013M11 gene	3.1
66	Mm.38192	schlafen 4	3.0
67	Mm.16224	guanylate cyclase activator 1a (retina)	3.0
68	Mm.280255	OVO homolog-like 1 (Drosophila)	3.0
69	Mm.71915	ESTs	3.0
70	Mm.379041	ESTs, Weakly similar to XLR MOUSE X-LINKED LYMPHOCYTE-REGULATED PROTEIN PM1 [M.musculus]	3.0
71	Mm.57574	B lymphocyte gene 1	3.0
72	Mm.38465	ESTs	2.9
73	Mm.15295	epoxide hydrolase 2, cytoplasmic	2.9
74	Mm.40394	ESTs	2.9
75	Mm.71921	ESTs	2.9
76	Mm.21571	ESTs, Weakly similar to PUTATIVE CELL DIVISION PROTEIN KINASE 2 HOMOLOG [Caenorhabditis elegans]	2.9
77	Mm.30128	ESTs	2.8
78	Mm.3264	TXK tyrosine kinase	2.8
79	Mm.100144	DNA segment, Chr 3, Nuffield Department of Surgery 3	2.8
80	Mm.431317	B-cell translocation gene 4	2.8
81	Mm.248606	RIKEN cDNA 1200004I24 gene	2.8
82	Mm.29975	RIKEN cDNA 1810003P21 gene	2.8
83	Mm.25861	ESTs	2.8
84	Mm.289244	RIKEN cDNA 2310016C19 gene	2.8
85	Mm.95479	2'-5' oligoadenylate synthetase-like	2.8
86	Mm.3506	arginase type II	2.7
87	Mm.38165	solute carrier family 27 (fatty acid transporter), member 2	2.7
88	Mm.248498	RAD17 homolog (S. pombe)	2.7
89	Mm.29816	programmed cell death 6 interacting protein	2.7
90	Mm.360540	RIKEN cDNA 1700001E04 gene	2.6
91	Mm.235081	BH3 interacting domain death agonist	2.6
92	Mm.8137	chromodomain helicase DNA binding protein 1	2.6
93	Mm.4481	interleukin 17 receptor A	2.6
94	Mm.69004	ESTs	2.6
95	Mm.25672	ESTs	2.6
96	Mm.34348	RIKEN cDNA 4930431L18 gene	2.6

97	Mm.130159	ESTs	2.5
98	Mm.34104	DNA segment, Chr 4, ERATO Doi 800, expressed	2.5
99	Mm.1137	integrin beta 2 (Cd18)	2.4
100	Mm.32139	ESTs	2.4
101	Mm.15962	poly (ADP-ribose) glycohydrolase	2.4
102	Mm.328086	ectodysplasin-A	2.4
103	Mm.265917	thyroid hormone receptor alpha	2.4
104	Mm.2904	zinc finger protein 216	2.4
105	Mm.31729	ESTs	2.4
106	Mm.236256	breast cancer 2	2.4
107	Mm.2581	Eph receptor A2	2.4
108	Mm.290183	RIKEN cDNA 2610015K05 gene	2.4
109	Mm.30630	ESTs	2.3
110	Mm.2093	snail homolog, (Drosophila)	2.3
111	Mm.39972	expressed sequence AI848218	2.3
112	Mm.44089	ESTs, Highly similar to KIAA0453 protein [H.sapiens]	2.3
113	Mm.333096	ATP-binding cassette, sub-family G (WHITE), member 2	2.3
114	Mm.33743	ESTs	2.3
115	Mm.170657	RIKEN cDNA 1110058E16 gene	2.3
116	Mm.299693	RIKEN cDNA 1110038J12 gene	2.3
117	Mm.38851	RIKEN cDNA 5830445O15 gene	2.3
118	Mm.68992	ESTs, Weakly similar to unknown [H.sapiens]	2.3
119	Mm.142856	LIM homeobox protein 2	2.3
120	Mm.276389	heme oxygenase (decycling) 1	2.2
121	Mm.549	interferon gamma receptor 1	2.2
122	Mm.31098	ESTs	2.2
123	Mm.32842	natural killer tumor recognition sequence	2.2
124	Mm.471306	imprinted and ancient	2.2
125	Mm.25201	ESTs	2.2
126	Mm.168789	cyclin-dependent kinase inhibitor 1C (P57)	2.2
127	Mm.2485	nuclear DNA-binding protein	2.2
128	Mm.34859	RIKEN cDNA 5033413D16 gene	2.2
129	Mm.324242	RIKEN cDNA 1810005K14 gene	2.2
130	Mm.26088	RIKEN cDNA 4930577M16 gene	2.2
131	Mm.387671	meiosis-specific nuclear structural protein 1	2.1
132	Mm.24755	ESTs	2.1
133	Mm.29616	ESTs	2.1
134	Mm.279116	RIKEN cDNA 2410004E01 gene	2.1

135	Mm.27059	ESTs	2.1
136	Mm.18263	gamma-glutamyltransferase-like activity 1	2.1
137	Mm.648	prion protein	2.1
138	Mm.58488	ESTs	2.1
139	Mm.385759	bone morphogenetic protein 6	2.1
140	Mm.115175	ESTs	2.1
141	Mm.31336	ESTs	2.1
142	Mm.4173	DEAD/H (Asp-Glu-Ala-Asp/His) box polypeptide 26	2.1
143	Mm.272748	RIKEN cDNA 4921524J17 gene	2.1
144	Mm.28940	RIKEN cDNA 5530401J07 gene	2.1
145	Mm.31436	myeloid ecotropic viral integration site-related gene 1	2.1
146	Mm.371552	Cd63 antigen	2.1
147	Mm.24383	ESTs, Weakly similar to unnamed protein product [M.musculus]	2.1
148	Mm.427266	RAR-related orphan receptor alpha	2.1
149	Mm.476828	RIKEN cDNA 1110025I09 gene	2.0
150	Mm.1273	retinoic acid receptor, gamma	2.0
151	Mm.24067	ESTs, Moderately similar to TATA binding protein associated factor [H.sapiens]	2.0
152	Mm.26688	thrombospondin 2	2.0
153	Mm.65655	ESTs, Weakly similar to I48668 zinc finger protein 51 - mouse [M.musculus]	2.0
